# Black gas, bright future: H_2_S based therapeutics for neurodegenerative disorders

**DOI:** 10.1016/j.neurot.2025.e00755

**Published:** 2025-09-24

**Authors:** Jordan L. Morris, Jordan J. Lee, Russell E. Morris, Jan Lj. Miljkovic

**Affiliations:** a MRC Mitochondrial Biology Unit, Keith Peters Building, Cambridge Biomedical Campus, University of Cambridge, CB2 0XY, UK; b Awen Oncology Ltd. Menai Science Park, Anglesey, LL60 6AG, UK; c Department of Medicine, University of Cambridge, Addenbrookes Hospital, Hills Road, Cambridge CB2 0QQ, UK; d EaStCHEM School of Chemistry, University of St Andrews, St Andrews, KY16 9ST, UK

**Keywords:** Gasotransmitters, Hydrogen sulfide (H₂S), Neurotherapeutics, Redox signaling, Protein persulfidation, Neurodegeneratio

## Abstract

From shaping Earth's earliest anoxic seas to quietly orchestrating cellular life today, hydrogen sulfide (H_2_S) has journeyed from ancient toxin to modern therapeutic candidate. Once abundant in Earth's primordial environment, H_2_S has reemerged as a critical endogenous gasotransmitter in modern biology. Within the central nervous system, H_2_S regulates redox homeostasis, mitochondrial bioenergetics, inflammatory signalling, and neuronal excitability. A key mechanism involves post-translational modification of protein cysteine residues (persulfidation), reactions with metal centres, and scavenging of reactive oxygen and nitrogen species, thereby influencing diverse cellular processes. Dysregulation of H_2_S metabolism, whether deficient or excessive, is increasingly implicated in neurodegenerative diseases such as Alzheimer's, Parkinson's, Huntington's disease, Down syndrome, and in stroke and traumatic brain injury. This review focuses on neuronal aspects of H_2_S biology and therapeutic relevance in these conditions. Restoration of H_2_S signalling in preclinical models improves cognitive and motor function, reduces neuropathology, and preserves mitochondrial integrity. Therapeutic innovation has produced a variety of H_2_S donors, including slow-releasing compounds, organelle-targeted agents, and emerging nanomaterial platforms such as polymer-based and metal–organic frameworks for precision CNS delivery. Natural compounds such as ergothioneine, a sulfur-containing antioxidant, are also gaining attention as potential modulators of endogenous H_2_S pathways. Future directions include integration of H_2_S therapies with genetic targeting tools and elucidation of their interactions with other gasotransmitters and gut–brain axis signalling. Although clinical trials remain limited, the convergence of donor chemistry, molecular biology, and delivery technologies positions H_2_S-based therapeutics as a promising frontier for treating neurodegeneration and acute neural injuries.

## Introduction: H_2_S, A homeostatic regulator in disguise

Cells exist in a state of dynamic equilibrium, continuously sensing and responding to fluctuations in their internal and external environments. This homeostatic balance is maintained through tightly regulated networks of signalling molecules that coordinate redox status, energy metabolism and adaptive gene expression [[1], [2], [3]]. Among these modulators is hydrogen sulfide (H_2_S), a small gaseous molecule that once billowed like black smoke from ocean bedrock during Earth's early history, shaping the euxinic environments from which life itself emerged [4]. Despite this primordial legacy, H_2_S was long dismissed as merely a toxic and foul-smelling chemical until its rediscovery as a key endogenous signalling molecule in modern biology. H_2_S is now recognised as a *bona fide* gasotransmitter alongside nitric oxide (NO) and carbon monoxide (CO), as these molecules permeate the cell membrane *via* passive diffusion without the requirement of a specific transporter [[5], [6], [7]]. H_2_S manifests its diverse biological effects through mechanisms such as posttranslational modification (PTM), reactions with metalloproteins, modulation of ion channels and redox buffering [8,9]. Its pleiotropic roles in stress adaptation, mitochondrial function and inflammation have positioned H_2_S as a compelling candidate for therapeutic exploitation, particularly in the context of neurological disease, where redox imbalance and metabolic dysfunction are prominent pathological features [10].

**Fig. 1:**
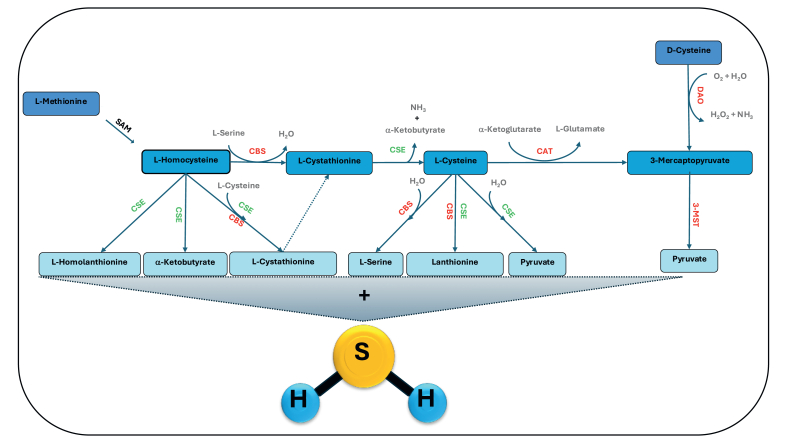
Canonical enzymatic pathways for endogenous hydrogen sulfide (H_2_S) production in mammals. The transsulfuration pathway involves the conversion of l-methionine to l-homocysteine *vi*a methionine recycling pathway and subsequently to l-cystathionine and l-cysteine *via* the enzymes cystathionine β-synthase (CBS) and cystathionine γ-lyase (CSE). l-cysteine serves as a substrate for both CBS and CSE, contributing directly to H_2_S generation. The alternative pathway involving cysteine aminotransferase (CAT) and 3-mercaptopyruvate sulfurtransferase (3-MST) utilizes l-cysteine or d-cysteine (following deamination by d-amino acid oxidase, DAO) to produce 3-mercaptopyruvate, a key intermediate for mitochondrial H_2_S production. The diagram illustrates the interconnected routes of transsulfuration and the contribution of each enzymatic step to H_2_S biosynthesis, emphasizing the roles of CBS, CSE, CAT, 3-MST, and DAO.

In mammals, enzymatic production of H_2_S occurs *via* two main distinct pathways (Fig. 1). The transsulfuration pathway, located primarily in the cytosol, involves cystathionine β-synthase (CBS) and cystathionine γ-lyase (CSE), which convert homocysteine and cysteine into H_2_S in a pyridoxal 5′-phosphate (PLP)-dependent manner. Separately, in the mitochondrial-associated pathway, cysteine aminotransferase (CAT) converts cysteine into 3-mercaptopyruvate (3-MP), which is then used by 3-mercaptopyruvate sulfurtransferase (3-MST) to generate H_2_S [11]. CBS is highly expressed in the brain (astrocytes and neurons of cortex, hippocampus, etc.), whereas CSE predominates in peripheral tissues but is also present in the CNS [12]. 3-MST, often working with cysteine aminotransferase (CAT), contributes to mitochondrial H_2_S production in neurons. A third minor pathway uses d-cysteine with d-amino acid oxidase and 3-MST [[13], [14], [15]]. These enzymes are regulated allosterically and transcriptionally, for instance CBS by S-adenosylmethionine and CO, and CSE by intracellular Ca^2+^ and transcription factors [[16], [17], [18], [19]]. An additional enzymatic route for H_2_S production has been recently identified involving human selenium-binding protein 1 (SELENBP1), which exhibits methanethiol oxidase activity analogous to that observed in methylotrophic bacteria. SELENBP1 catalyses the oxidative degradation of methanethiol, resulting in the formation of H_2_S, hydrogen peroxide (H_2_O_2_), and formaldehyde [20]. Alternatively, H_2_S can be generated through a non-enzymatic reaction involving l-cysteine, ferric iron (Fe^+3^), and vitamin B_6_, as shown in a study investigating this alternative pathway [21]. However, the actual contribution of this non-enzymatic route to the total circulating H_2_S pool remains uncertain and is open to question, particularly in light of the slow kinetics of the reaction and the low amounts of H_2_S detected even under supraphysiological or physiological substrate conditions. Finally, gut microbiota constitutes the important source of H_2_S in mammals, contributing to both systemic and local sulfide pools [22,23]. Recent studies have highlighted the microbial production of H_2_S not only as a modulator of intestinal homeostasis but also as a potential driver of pathological processes, including tumorogenesis and neurodegeneration [[24], [25], [26]].

In biological systems, H_2_S exists in equilibrium with its deprotonated form, hydrosulfide (HS^−^), with a pKa of ∼6.9 and at physiological pH (∼7.4), HS^−^ is the dominant species, while some H_2_S remains in its neutral, membrane-permeable form [27]. The fully deprotonated sulfide ion (S^2−^), with a pKa >12, is not present under normal conditions and only forms in highly alkaline environments. The hydrosulfide anion (HS^−^) is primarily responsible for the biological actions of H_2_S, which can be broadly categorised into three main mechanisms: redox-dependent post-translational modifications; interactions with metal centres and metalloproteins; and the scavenging of reactive oxygen species (ROS) as well as reactive nitrogen species (RNS) [27].

The primary route for H_2_S catabolism is mitochondrial oxidation [28,29]. This process begins with oxygen-independent activity of sulfide:quinone oxidoreductase (SQR), a mitochondrial inner membrane protein, which initiates the oxidation of H_2_S by transferring electrons from H_2_S to the oxidised form of coenzyme Q (CoQ), thereby feeding electrons into the respiratory chain and contributing to ATP synthesis [30]. During this reaction, SQR converts H_2_S into a protein-bound persulfide intermediate (SQR-SSH). The persulfide generated on SQR can then follow transfer of sulphur atoms from SQR-SSH to sulphite (SO_3_^2−^), leading to the formation of thiosulfate (S_2_O_3_^2−^) in a reaction involving thiosulfate sulfurtransferase (TST), which can further transfer sulphur to glutathione (GSH), producing glutathione persulfide (GSSH) and regenerating SO_3_^2−^ [31,32]. Alternatively, sulphur atom from SQR-SSH can be transferred directly to GSH to form GSSH, which is subsequently oxidised by persulfide dioxygenase (ETHE1) to yield SO_3_^2−^ [33]. The resulting SO_3_^2−^ is then oxidised to sulphate (SO_4_^2−^) by sulphite oxidase (SO) and excreted renally [34]. Additionally, rhodanese (Rhd) can catalyse the transfer of sulphur atom from GSSH to SO_3_^2−^ producing S_2_O_3_^2−^, much of which is ultimately converted to SO_4_^2−^ by the sequential action of thiosulfate reductase (TR) and SO [35].

### Regulation of canonical pathways

One of the principal mechanisms by which H_2_S exerts its biological effects is through protein persulfidation (also known as *S*-sulfhydration), a post-translational modification involving the addition of a sulphur atom (S) to the thiol group (-SH) of cysteine residues, forming a persulfide (-SSH). This redox-based modification, often compared to *S*-nitrosylation (nitric oxide-dependant PTM), can significantly influence protein activity, stability, and cellular signalling dynamics [27,36]. Persulfidation often enhances enzyme or protein activity or modifies how proteins interact with partners [37]. For example, sulfhydration of the glycolytic enzyme glyceraldehyde-3-phosphate dehydrogenase (GAPDH) has been shown to greatly increase its activity, and persulfidation of actin promotes actin polymerisation, affecting cytoskeletal dynamics [36].

Early work by Kimura and colleagues established that H_2_S enhances N-methyl-d-aspartate receptor (NMDAR) currents and promotes hippocampal long-term potentiation (LTP), a cellular correlate of learning and memory [38]. More recent studies have revealed that H_2_S can induce persulfidation of serine racemase (SR), an enzyme critical for the synthesis of d-serine, a co-agonist of NMDARs. This modification upregulates SR activity, thereby potentiating NMDAR signalling and further promoting LTP [39]. In addition, H_2_S and polysulfides have been shown to increase the release of neurotransmitters including γ-aminobutyric acid (GABA), d-serine, and glutamate, leading to transient inhibition of NMDAR activity. These effects were found to depend on the activity of transient receptor potential ankyrin 1 (TRPA1) channels and 3-MST, providing further mechanistic insight into how sulphur signalling modulates synaptic plasticity. Notably, this pathway has emerging implications in behavioural regulation and psychiatric phenotypes [40].

In parallel, H_2_S modulates various ion channels, which alters neuronal and glial excitability as well as vascular tone. A well-characterised target is the ATP-sensitive K^+^ channel (K_ATP) found in many cell types. H_2_S reacts with a conserved cysteine on the channel's Kir6.x subunits (e.g., Cys43 on Kir6.1), persulfidating that site [41]. This prevents ATP from binding and closing the channel, thereby keeping the K^+^ channel open. The result is an efflux of K^+^ ions that hyperpolarises the cell membrane. In neurons, this hyperpolarisation can reduce firing rates, and in smooth muscle (such as in cerebral arteries) causes relaxation and vasodilation [42]. H_2_S similarly activates certain Ca^2+^-activated K^+^ channels, which can modulate calcium-dependent electrical activity and signalling in both neurons and astrocytes [43,44]. Additionally, application of H_2_S, either as sodium hydrosulfide (NaHS) or *via* the slow-releasing donor GYY4137 to primary cultured rat hippocampal neurons was shown to induce persulfidation of a critical cysteine residue (Cys73) on the voltage-gated potassium channel Kv2.1, resulting in channel inhibition and a consequent enhancement of neuronal excitability [45]. Through these actions on ion channels, H_2_S generally has an inhibitory effect on over-excitation, though in some contexts (for example, by modulating Ca^2+^ flux in astrocytes) it may indirectly facilitate excitatory signalling. H_2_S also affects the varieties of voltage dependant Ca^2+^ channels (VDCC) [46,47], such as L-type Ca^2+^ channel (LTCC) in heart and muscle [48,49], N, P/Q and T-type Ca^2+^ channels expressed in neuronal tissue [46,50]. Importantly, these canonical signalling mechanisms of persulfidation of proteins and the gating of ion channels are unique to H_2_S, even as they conceptually parallel the mechanisms used by NO and CO [8,51].

### Redox regulation and non-canonical pathways

Beyond its direct effects on proteins and channels, H_2_S profoundly influences cellular redox pathways and survival signalling. One key role of H_2_S is as an antioxidant. It can directly scavenge ROS and RNS (e.g. peroxynitrite), whilst also elevating the cell's endogenous antioxidant capacity [52,53]. A prime example of the latter is H_2_S's interaction with the Keap1 Nrf2 system. Under basal conditions, the transcription factor Nrf2 is kept inactive in the cytosol by its inhibitor Keap1. H_2_S can persulfidate Keap1 (for instance at cysteine-151), which weakens the Keap1 Nrf2 binding [54]. This allows Nrf2 to translocate into the nucleus and induce a suite of cytoprotective genes including those coding for glutathione synthesis (e.g., glutamate-cysteine ligase), NAD(P)H:quinone oxidoreductase 1 (NQO1), and haeme oxygenase-1 (HO-1). Through Nrf2 activation, H_2_S ramps up the production of glutathione and other antioxidants, thereby fortifying neurons against oxidative stress and ischemic injury [55,56].

H_2_S also modulates signalling pathways involved in inflammation and cell survival, acting in what might be considered “non-canonical” fashions (i.e., not purely through persulfidation). For example, during inflammatory stress, H_2_S can influence the NF-κB pathway. Tumour necrosis factor-α (TNF-α) stimulation is known to increase Sp1-mediated transcription of CSE, leading to higher H_2_S production^45^. The additional H_2_S then persulfidates the p65 subunit of NF-κB. Persulfidation of p65 enhances its binding to the coactivator RPS3, which skews NF-κB towards activating gene programs that are anti-apoptotic and pro-survival, rather than pro-inflammatory [57]. In essence, H_2_S can tip the balance of NF-κB signalling to favour cell survival under stress conditions. This is one example of how H_2_S intersects with major regulatory networks. Other studies have shown H_2_S can modulate pathways like PI3K/Akt and MAPK/ERK [58,59], further pointing its potential in neural cells.

### Epigenetic interactions

Emerging evidence indicates that H_2_S signalling can extend to epigenetic regulation of gene expression [60,61]. One avenue is *via* modification of chromatin-modifying enzymes. H_2_S or H_2_S-donating molecules have been found to inhibit certain DNA methyltransferases (DNMTs) and histone deacetylases (HDACs) in experimental systems^48^. By modulating these enzymes, H_2_S can lead to changes in DNA methylation patterns and histone acetylation status, resulting in the upregulation of protective genes or the silencing of deleterious ones. For instance, H_2_S donor treatment in neuronal cultures has been associated with decreased global DNA methylation and increased acetylation of histone tails, changes that correlate with the expression of genes promoting cell survival and neuroplasticity [62]. Recent evidence suggests that H_2_S may influence epigenetic regulation in neurodegeneration. In a rat model of Parkinson's disease (PD) induced by 6-hydroxydopamine (6-OHDA), H_2_S treatment attenuated disease-associated phenotypes, including reductions in dopamine, its metabolite 3,4-dihydroxyphenylacetic acid (DOPAC), and histone deacetylase (HDAC) activity [63]. These findings point towards a neuroprotective mechanism involving the modulation of histone acetylation pathways.

Another epigenetic layer involves non-coding RNAs. H_2_S has been linked to the regulation of several microRNAs (miRNAs) that, in turn, affect H_2_S production or action [64,65]. Conversely, some miRNAs respond to alterations in H_2_S levels [66,67]. A notable example is miR-125b-5p, a microRNA that directly targets CBS mRNA. In models of hypoxic neuronal injury, overexpression of miR-125b-5p leads to downregulation of CBS, reducing H_2_S synthesis and worsening cell damage [67]. Inhibition of miR-125b-5p, on the other hand, prevents the loss of CBS and helps maintain H_2_S production, thereby protecting neurons under stress. In other contexts, H_2_S has been shown to modulate the levels of long non-coding RNAs such as MALAT1, as well as miRNAs like miR-30c and miR-485-5p, which have been implicated in H_2_S-driven protective effects during spinal cord ischemia and in models of neuronal apoptosis [[68], [69], [70]]. Collectively, these findings suggest that H_2_S is woven into a complex epigenetic network, influencing epigenetic regulators and is, itself subject to regulation by non-coding RNAs, ultimately impacting gene expression programs that determine neuronal fate.

### Developmental roles of H_2_S

H_2_S is not only important for maintaining adult brain function but also plays significant roles during neurodevelopment. The expression of CBS (the dominant H_2_S-producing enzyme in the brain) is highest in the early postnatal period, corresponding with phases of intense neurodevelopmental remodelling [12]. Studies indicate that H_2_S promotes neurite outgrowth and axonal elongation, suggesting a guidance role for growing neurons [71]. It also appears to facilitate synaptogenesis i.e. the formation of new synapses, possibly through interactions with calcium signalling pathways and the activation of growth-related kinases like MAPK/ERK [72,73]. These developmental effects of H_2_S are consistent with observations in cell culture: adding an H_2_S donor to cultured neurons enhances the formation of dendritic spines and synaptic connections [71].

H_2_S may additionally protect immature neurons at a life stage when oxidative stress can be particularly harmful. Around the time of birth and in early brain development, neurons experience high metabolic demands and generate significant levels of reactive oxygen species. H_2_S helps counteract this oxidative stress, thereby safeguarding developing neural circuits [12]. In animal models, inhibiting H_2_S production during development (for example, using a CBS inhibitor) has led to neurodevelopmental deficits, including impaired hippocampal neurogenesis and cognitive dysfunction later in life [74]. Conversely, mice genetically deficient in certain H_2_S-producing enzymes show altered brain morphology and behaviour. While research in humans is still nascent, some studies have speculated that aberrant H_2_S metabolism might be a contributing factor in developmental disorders such as autism spectrum disorder [75,76]. Although direct evidence for this in patients is limited, the developmental necessity for balanced H_2_S signalling is clear from animal studies.

## Dysregulation of H_2_S in neurological disease

### Alzheimer's disease (AD)

Growing evidence suggests that endogenous H_2_S is deficient in Alzheimer's disease. Post-mortem analyses of AD brains often show reduced expression of CBS (the primary H_2_S-generating enzyme in the brain) and correspondingly lower H_2_S levels compared to age-matched healthy brains [77]. This deficit in H_2_S correlates with hallmarks of Alzheimer's pathology: increased oxidative damage, accumulation of amyloid-β plaques, and hyperphosphorylation of tau protein. Insufficient H_2_S deprives neurons of critical redox buffering and signalling support, potentially accelerating synaptic dysfunction [77]. Supporting this view, experiments in AD animal models have demonstrated that replenishing H_2_S can ameliorate disease features. Treatment of APP-transgenic mice with H_2_S donors (such as NaHS or the slow-releasing GYY4137) led to improvements in memory tasks and a reduction in soluble Aβ levels and plaque deposition [77,78]. These benefits are attributed to H_2_S restoring glutathione content, reducing neuroinflammation, and re-activating pro-survival pathways that are otherwise impaired in the Alzheimer's brain.

Recent work by further clarified the role of endogenous H_2_S in Alzheimer's disease by demonstrating that CSE-dependent sulfide production leads to the persulfidation of glycogen synthase kinase 3β (GSK3β), a key regulator of tau phosphorylation [79]. This redox modification suppresses GSK3β activity, thereby limiting tau aggregation, a hallmark of AD pathology. Moreover, *in vivo* administration of H_2_S donors improved cognitive function and reduced tau-related neuropathology in transgenic AD mouse models, reinforcing the therapeutic promise of targeting H_2_S signalling in tauopathies and neurodegeneration.

### Parkinson's disease (PD)

In Parkinson's disease, a loss of H_2_S signalling has been linked to the degeneration of dopaminergic neurons. One crucial H_2_S-sensitive target in these neurons is the ubiquitin E3 ligase parkin, which helps clear misfolded proteins. Under normal conditions, parkin is persulfidated by H_2_S at specific cysteine residues (Cys59, Cys95, and Cys182) [80]. This persulfidation enhances parkin's enzymatic activity, promoting the removal of potentially toxic proteins such as oxidised or misfolded α-synuclein. In PD patients, studies have found that parkin in the substantia nigra and striatum is under-persulfidated, which coincides with a buildup of protein aggregates that parkin would normally help degrade [80]. A deficiency in H_2_S whether due to lower CSE/CBS expression or increased consumption of H_2_S by oxidative stress could thus impair this protective protein quality-control mechanism. Consistent with this, H_2_S donors have shown neuroprotective effects in Parkinson's models: administration of H_2_S-releasing compounds in MPTP-treated rats preserves nigral neuron counts and improves motor performance [81]. These outcomes involve H_2_S maintaining mitochondrial function (preventing the loss of complex IV activity and ATP depletion that occur in PD models) and limiting neuroinflammatory responses (such as microglial activation). The PD case exemplifies how a drop in H_2_S bioavailability can contribute to neurodegenerative processes, and conversely, how boosting H_2_S can intervene in those processes.

### Huntington's disease (HD)

Huntington's disease is another neurodegenerative disorder where H_2_S dysregulation has been observed, albeit *via* a different mechanism. In models of HD, the mutant huntingtin protein (mHTT) aberrantly sequesters various transcription factors, including Specificity Protein 1 (Sp1) [82]. Sp1 is known to regulate the expression of CSE. When Sp1 is trapped by mHTT, CSE expression in affected neurons (especially in the striatum) is markedly reduced. The downstream effect is a decrease in H_2_S production in those cells. This loss of H_2_S is believed to exacerbate the oxidative stress and metabolic impairment characteristic of Huntington's disease. Normally, H_2_S would support mitochondrial function and antioxidant defences, but in its absence, neurons become more susceptible to damage from reactive oxygen species and impaired energy metabolism [82]. While direct supplementation of H_2_S in HD models has been less studied than in AD or PD, the mechanistic link between mHTT, Sp1, and CSE provides a rationale for exploring H_2_S-elevating therapies in Huntington's disease as well.

### Down syndrome (DS)

Down syndrome (trisomy 21) presents a unique scenario in which H_2_S is in excess rather than deficient [83]. People with Down syndrome have an extra copy of the CBS gene, leading to overproduction of H_2_S [84]. Studies have found that individuals with Down syndrome (DS) tend to have elevated levels of H_2_S metabolites; for example, they excrete higher amounts of thiosulfate in urine, reflecting increased H_2_S turnover [84]. In the brain, overactive CBS could result in supraphysiological H_2_S levels in certain regions. While physiological levels of H_2_S are protective, excessive concentrations have been shown to impair ATP production by inhibiting mitochondrial respiration, particularly through inhibition of Complex IV [85], as well as potentially disrupting other metabolic processes in cultured primary fibroblast from DS patients. Some researchers hypothesize that chronically elevated H_2_S in Down syndrome could contribute to the developmental and cognitive abnormalities observed in this condition [84]. Indeed, in a novel rat model of DS induced by duplication of a selected region of chromosome 20 (parts of the region from Umodl1 to Prmt2) containing the CBS gene, corresponding elevated H_2_S levels were shown to disrupt gamma-frequency brain electrical activity and suppress the expression of key synaptic proteins, including postsynaptic density protein 95 (PSD-95) and synaptophysin [86]. The same research group recently extended these findings in a mouse model employing a similar “minimalistic” duplication of a chromosome region containing the CBS gene, demonstrating that elevated brain H_2_S levels impair ER stress responses and reduce autophagy capacity [87]. Notably, this study also reported sex-specific effects, with female animals more affected, and revealed that excess H_2_S exerts complex dysregulatory effects on multiple metabolic pathways including amino acid, nucleotide, endocannabinoid, and carbohydrate metabolism ultimately leading to deficits in cognitive functions such as spatial learning and recognition memory. Notably, in both studies from this research group, administration of aminooxyacetic acid (AOAA), an inhibitor of CBS, reversed the observed pathological phenotype, further supporting the specificity of a CBS–H_2_S axis in driving neuropathological changes [86,87]. Collectively, these findings underscore that H_2_S homeostasis requires careful balance, as both deficiency and excess of H_2_S can have detrimental effects on the brain.

### Stroke and traumatic brain injury

Acute injuries to the brain, such as ischemic stroke and traumatic brain injury (TBI), are accompanied by disruptions in H_2_S signalling. In the case of stroke (especially during the reperfusion phase after ischemia), endogenous H_2_S levels in the brain can drop [[88], [89], [90]]. This decline is thought to worsen outcomes because H_2_S is needed to induce vasodilation in reperfusing vessels and to neutralise the burst of reactive oxygen species and subsequent oxidative damage that accompanies reperfusion [[91], [92], [93]]. Experiments in rodent stroke models have shown that administering an H_2_S donor either just before or during reperfusion significantly reduces brain damage, infarct volumes are smaller with less neuronal death. H_2_S helps maintain blood-brain barrier integrity and cerebral blood flow in these settings, likely due to its combined vasodilatory and antioxidant effects [94,95].

In TBI, a somewhat similar pattern emerges: trauma can impair the activity of H_2_S-producing enzymes, leading to a transient sulfide deficit when the tissue is under severe oxidative and inflammatory stress [96]. Animal studies of TBI have demonstrated that H_2_S-based treatments (such as infusing Na_2_S or an H_2_S-releasing compound) result in reduced brain edema, decreased release of pro-inflammatory cytokines, and improved neurological recovery [97]. H_2_S appears to preserve mitochondrial function in traumatized neurons and may inhibit the activation of apoptosis cascades post-injury. These findings suggest that timely restoration of H_2_S following acute brain insults can mitigate secondary damage and improve healing.

### Other neurological conditions

Beyond the major disorders above, altered H_2_S metabolism has been noted in several other neurological conditions. Patients with amyotrophic lateral sclerosis (ALS), for instance, have shown abnormal levels of sulphur compounds in blood and cerebrospinal fluid in some studies, hinting at a possible involvement of H_2_S signalling in motor neuron disease [[98], [99], [100]]. Similarly, in certain forms of epilepsy, researchers have observed changes in the expression of H_2_S-synthesizing enzymes in the hippocampus, suggesting H_2_S might modulate seizure susceptibility (perhaps through its effects on ion channels and neurotransmitter release [101]. While these connections are still being explored, it is clear that H_2_S's influence extends broadly. In conditions like ALS, H_2_S could be affecting pathways of excitotoxicity or mitochondrial health in motor neurons. In epilepsy, H_2_S might be regulating neuronal excitability and inflammation in the epileptic focus. Overall, whether it is chronic diseases or acute injuries, the common theme is that a deviation from normal H_2_S homeostasis either a shortfall or an excess can contribute to neural dysfunction, whereas correcting that imbalance has potential therapeutic value.

## Therapeutic potential of H_2_S modulation

The recognition of H_2_S's neuroprotective effects has spurred interest in therapeutic strategies to modulate H_2_S levels in the brain. Broadly, the goal is to restore optimal H_2_S signalling in disease states, either by supplementing H_2_S using donor compounds or by enhancing the activity of endogenous H_2_S-producing enzymes. A wide array of preclinical studies lends support to this approach. Even in models of chronic neurodegeneration like Huntington's disease, preliminary studies suggest that boosting H_2_S can ameliorate mitochondrial abnormalities and cell death [82]. Likewise, in rodent models of ischemic stroke, infusion of an H_2_S donor during the reperfusion phase reduced the size of the brain infarct and improved functional recovery, presumably by limiting oxidative damage and preserving blood flow [102,103].

Mechanistically, H_2_S exerts its beneficial effects through several converging pathways. As discussed earlier, H_2_S enhances antioxidant defences (*via* glutathione and Nrf2-dependent enzymes) and curtails harmful ROS accumulation. It also modulates inflammatory signalling, for instance, H_2_S can suppress the activation of microglia and astrocytes, resulting in lower production of pro-inflammatory cytokines in the brain milieu [104]. Concurrently, H_2_S activates pro-survival signalling cascades. It has been shown to upregulate factors like brain-derived neurotrophic factor (BDNF) and to activate kinases such as Akt, which promote cell survival and neuroplasticity [105,106]. In some contexts, H_2_S persulfidation of key metabolic enzymes helps maintain energy production, giving stressed neurons a better chance to survive. Interestingly, All these actions position H_2_S as a broad-spectrum protective agent in the CNS.

It is also informative to consider what happens when H_2_S production is inhibited in otherwise healthy systems. Pharmacological blockers of H_2_S synthesis (such as propargylglycine, which inhibits CSE, or aminooxyacetic acid, an inhibitor of CBS) tend to worsen outcomes in models of disease and can even produce deleterious effects by themselves [107,108]. Genetic knockout studies align with this, where mice lacking CSE or CBS have heightened susceptibility to oxidative damage, inflammation, and hypertension [108,109]. Conversely, cardiac-specific CSE overexpression in rats was associated with an increase in the rate of H_2_S production, and had smaller infarct size following myocardial infarction when compared to their wildtype controls [110]. The protective effects of H_2_S was later demonstrated to be mediated through Nrf2 signalling [55]. These findings underscore that basal H_2_S production is a part of the body's natural defence repertoire. Therefore, a therapeutic strategy aiming to increase H_2_S is essentially augmenting an endogenous protective system.

Given H_2_S's interactions with other gasotransmitters, there are intriguing possibilities for combination therapies. H_2_S and NO, for example, both promote vasodilation but *via* partly distinct mechanisms. H_2_S opens K_ATP channels while NO stimulates cGMP production. Studies have found that H_2_S can enhance NO signalling by inhibiting phosphodiesterases [111] (which break down cGMP), suggesting a synergistic effect on blood vessels and possibly neurons. This synergistic interplay between endogenously produced and colocalized H_2_S and NO has been demonstrated in studies showing that their reaction product, nitroxyl (HNO), activates TRPA1 channels in a redox-sensitive manner (*via* formation of intramolecular disulfide bond). Subsequently, this triggers the release of calcitonin gene-related peptide (CGRP), a potent vasodilator, thereby modulating the neurovascular and endocrine signalling axis [112]. Additionally, H_2_S can help mitigate some toxic aspects of NO and *vice-versa*; their reaction product, a nitrosothiol-based specie thionitrous acid (HSNO), perthionitrous acid (SSNO^−^) or nitroxyl (HNO), may carry its own signalling functions [27,[112], [113], [114]]. In theory, a therapy that provides both NO and H_2_S (either *via* two separate donors or a single hybrid donor molecule) could yield additive benefits, such as improved cerebral blood flow coupled with robust antioxidant protection. While such combination approaches are still largely experimental, they represent a frontier of neurotherapeutic research spurred by the growing understanding of gasotransmitter biology.

Bolstering H_2_S signalling is emerging as a promising avenue to combat neurodegenerative diseases and acute neural injuries. By counteracting oxidative stress, modulating inflammation, preserving mitochondrial function, and engaging cell survival programs, H_2_S can intervene in multiple steps of neuronal injury cascades. The challenge moving forward is translating these multifaceted benefits into safe and controlled therapies for humans.

## H_2_S based neurotherapeutics: Chemical classes & delivery strategy

### Chemical classes and delivery strategies

**Fig. 2:**
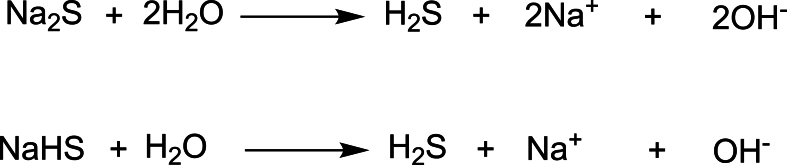
Chemical structures and hydrolysis-based release mechanism of hydrogen sulfide (H_2_S) from disodium sulfide (Na_2_S) and sodium hydrogen sulfide (NaHS). Both salts liberate H_2_S upon dissolution in aqueous solution through simple hydrolysis.

Given H_2_S's broad neuroprotective roles, therapeutic strategies increasingly aim to restore or enhance H_2_S signalling in disease contexts. In animal models, administration of synthetic H_2_S donors or upregulation of endogenous biosynthetic enzymes (e.g., CSE, CBS or 3-MST) has been shown to mitigate neuropathology. A variety of chemical H_2_S donors have been developed to control H_2_S release kinetics and targeting [[115], [116], [117], [118]]. Rapid-release donors include sulfide salts (Fig. 2) such as sodium hydrosulfide (NaHS) and disodium sulfide (Na_2_S) which dissociate in solution to yield H_2_S but cause a transient high spike. Slow-releasing donors are designed to provide a sustained and stable release of H_2_S over several hours, avoiding toxic peaks and allowing H_2_S levels to rise gradually and persist longer *in vivo.* NaHS solutions typically contain significant impurity levels (up to 40 ​%) and rapidly degrade in aqueous systems, which underscores the importance of careful experimental design and the inclusion of appropriate controls to accurately attribute observed effects to H_2_S. It is also important to note that GYY4137 (Section 4. 3) is not a pure H_2_S source; it contains equimolar morpholine and is commonly synthesized in dichloromethane, a solvent that may yield carbon monoxide production *in vivo*. These elements may contribute to observed biological effects, making it essential to consider the full chemical profile of the donor.

Therefore, to obtain clarity throughout this review, we refer to the biological effects attributed to H_2_S donors with the understanding that these effects may derive not solely from H_2_S itself, but also from donor-specific pharmacological properties, their parent molecules, by-products, or metabolites. We use the term “H_2_S” as a functional shorthand, while recognizing this complexity.

### Inorganic salts (NaHS & Na_2_S)

In Alzheimer's disease models, chronic administration of H_2_S donors, such as the fast-releasing sodium hydrosulfide (NaHS) salt or Tabiano's spa-water, a mineral water with the highest concentration of H_2_S among all natural springs in Europe, has been shown to improve cognitive function and slow the progression of pathology [77]. In rat models of AD induced either by β-amyloid peptide injection or streptozotocin exposure, both short- and long-term treatments significantly delayed symptom onset and mitigated cognitive impairments, including deficits in memory and learning. Similar benefits were observed in transgenic 3xTg-AD mice, where these treatments reduced expression of pro-apoptotic and pro-inflammatory proteins, lowered levels of phosphorylated tau, and decreased amyloid-β plaque burden.

In experimental models of Parkinson's disease, H_2_S supplementation using NaHS protected dopaminergic neurons in rats with 6-hydroxydopamine (6-OHDA)-induced pathology [81]. In these studies, NaHS-treated animals exhibited reduced markers of neurotoxicity, including preservation of tyrosine hydroxylase-positive cells and decreased levels of malondialdehyde, nitric oxide, and TNF-α. Interestingly, NaHS also prevented the onset of motor dysfunction in the rotenone-induced model of Parkinson's disease, further supporting its neuroprotective potential.

A recent study has further highlighted the neuroprotective potential of gaseous H_2_S in experimental Parkinson's disease [119]. In this model, Parkinsonian pathology was induced by administration of the neurotoxin MPTP, after which animals were treated *via* inhalation of 40 ​ppm H_2_S gas for 8 ​h daily over a one-week period. This exposure prevented the loss of tyrosine hydroxylase-positive neurons and ameliorated locomotor deficits. In addition, animals treated with gaseous H_2_S displayed enhanced expression of antioxidant response elements such as glutamate–cysteine ligase (GCL) and haeme oxygenase-1 (HO-1), alongside reductions in pro-inflammatory and pro-apoptotic markers.

Administration of NaHS has also shown protective effects in models of traumatic brain injury (TBI). In a mouse model where TBI was induced *via* craniotomy and mechanical trephine-induced stress, pre-treatment with NaHS resulted in reduced brain edema, improved locomotor performance, and enhanced cognitive outcomes as assessed by the Morris water maze test [120]. Mechanistically, NaHS pre-treatment attenuated the expression of pro-apoptotic markers, including cleaved caspase-3 and Bcl-2, while preserving autophagy flux in the injured brain.

These examples highlight a general trend: when H_2_S is restored towards normal physiological levels in a damaged or diseased brain, multiple aspects of pathology (oxidative stress, inflammation, metabolic failure) tend to improve.

### Synthetic organic molecules

**Fig. 3:**
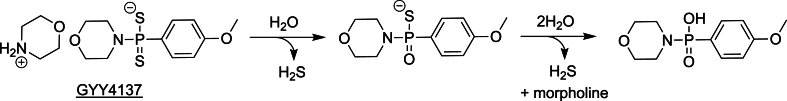
Chemical structure and proposed H_2_S release mechanism of GYY4137 (morpholine (4-methoxyphenyl)(morpholino)phosphinodithioate). GYY4137 is a slow-releasing hydrogen sulfide donor that undergoes stepwise hydrolysis under aqueous conditions, liberating H_2_S and morpholine as by-products.

Synthetic organic H_2_S donors have been developed to achieve more controlled release profiles. Among the various H_2_S donors developed to date, GYY4137 (morpholine (4-methoxyphenyl)(morpholino)phosphinodithioate) (Fig. 3) designed and introduced by the Whiteman group remains the most extensively studied slow-releasing donor. This compound undergoes spontaneous hydrolysis under physiological conditions, gradually liberating hydrosulfide (HS^−^) ions along with its hydrolytic inactive byproducts [[121], [122], [123]]. GYY4137 exhibits neuroprotective effects in Alzheimer's disease models by promoting the sulfhydration of glycogen synthase kinase 3β (GSK3β), thereby inhibiting tau hyperphosphorylation, a critical step in neurofibrillary tangle formation. In the context of Alzheimer's disease, an H_2_S-releasing memantine prodrug has been proposed as a next-generation therapeutic agent, potentially combining NMDAR antagonism with redox modulation [124]. Similarly, the l-Dopa derivative ACS84, engineered to release H_2_S, exerts therapeutic effects in a rat model of 6-hydroxydopamine-induced Parkinson's disease, preserving dopaminergic neurons and improving motor performance [125].

Other classes of H_2_S-releasing compounds, although primarily investigated outside of neurological contexts, include hybrid drugs in which conventional therapeutics have been chemically conjugated to H_2_S-donating moieties. Examples include NSAIDs such as H_2_S–diclofenac (ACS-15) and nitrate-based hybrids, designed to integrate anti-inflammatory effects with controlled H_2_S delivery.

### Targeted H_2_S delivery

The development of organelle-targeted therapeutics has opened new avenues for spatially precise drug delivery, with mitochondria emerging as a particularly compelling target for H_2_S-based interventions due to their pivotal role in cellular redox regulation and vulnerability in disease contexts.

Mitochondrial injury is a central event in both acute ischemic episodes (e.g. stroke) and chronic neurodegenerative conditions [126]. In ischemia-reperfusion (I/R) injuries, the abrupt restoration of blood flow to oxygen-deprived tissue sets off a surge of redox-active species (e.g., superoxide (O_2_^.-^) and peroxynitrite (ONOO^.-^) anion radicals, hydrogen peroxide (H_2_O_2_), hydroxyl radical (^.^OH), epoxyallylic peroxyl radicals (OLOO^·^)) that compromise mitochondrial integrity, leading to the initiation of cell death programs [3]. This cascade resembles the degenerative processes seen in disorders such as Parkinson's and Huntington's disease, where proteostasis collapse and mitochondrial distress accelerate neuronal loss. In both contexts, damaged mitochondria act as amplifiers of cellular stress, contributing to secondary injury and long-term functional decline [126]. Among the signalling molecules involved, hydrogen sulfide (H_2_S) presents a paradox: while protective at low levels, it becomes detrimental when dysregulated, particularly within mitochondria [127]. These mechanistic overlaps underscore the mitochondria as a key therapeutic target in both acute and chronic neuropathology [[128], [129], [130]]. Insights from ischemia/reperfusion models where modulating H_2_S levels can mitigate mitochondrial damage have prompted the development of strategies that precisely deliver H_2_S to subcellular compartments [131]. Such targeting is achieved by conjugating H_2_S-releasing chemotypes (“warheads”) to carrier groups (e.g., lipophilic cation) that accumulate selectively in the organelle. This strategy enables precise dosing, enhances therapeutic potency, and limits off-target effects.

The most widely explored strategy for delivering therapeutic cargo to mitochondria involves the use of lipophilic organic molecules bearing a positively charged moiety that enables selective, membrane potential (ΔΨm)-dependent accumulation within the mitochondrial matrix. Among these, the triphenylphosphonium (TPP^+^) cation has become the most frequently used mitochondrial targeting scaffold, due to its strong cationic-based uptake into mitochondria driven by the negative membrane potential across the inner mitochondrial membrane [132].

**Fig. 4:**
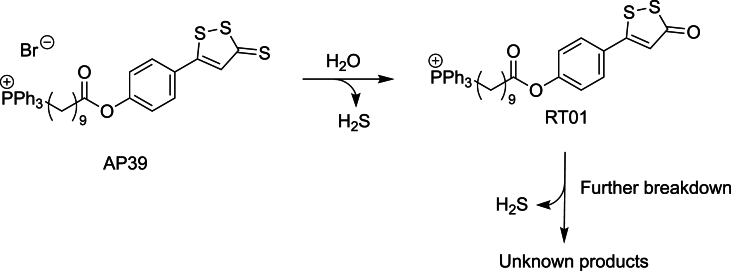
Chemical structure of the mitochondria-targeted H_2_S donor AP39 ([(10-oxo-10-(4-(3-thioxo-3H-1,2-dithiol-5-yl)phenoxy)decyl)triphenylphosphonium bromide]) and its proposed mechanism of hydrogen sulfide release under aqueous conditions. Hydrolysis of the dithiolthione moiety generates H_2_S, yielding the intermediate RT01, which undergoes further decomposition to release additional H_2_S and form unidentified breakdown products.

The first mitochondria-targeted H_2_S donor employing this strategy AP39 [(10-oxo-10-(4-(3-thioxo-3H-1,2-dithiol-5yl)phenoxy)decyl) triphenylphosphonium bromide] (Fig. 4), was developed by the Whiteman group [133] and it consists of a TPP ​^+^ ​moiety linked *via* a ten-carbon aliphatic chain to ADT-OH (5-(4-hydroxyphenyl)-3H-1,2-dithiole-3-thione), a natural garlic-derived H_2_S-releasing chemotype. This design enables spatially restricted H_2_S release within mitochondria, enhancing cytoprotective efficacy while limiting systemic toxicity [134].

In the context of neurotherapeutics, AP39 remains the most extensively studied mitochondria-targeted H_2_S donor. In a seminal study using a murine model of cardiac arrest followed by cardiopulmonary resuscitation (CPR), AP39 administered 2 ​min prior to reperfusion significantly improved neurological outcomes, preserved mitochondrial function, and enhanced post-intervention survival [135]. Recent independent studies further support the neuroprotective potential of AP39 in models of brain ischemia and hypoxia-reperfusion injury. In a rat model of middle cerebral artery occlusion (MCAO), a seven-day pretreatment with AP39 conferred significant ischemic tolerance, reducing infarct size and improving outcomes [136]. Separately, in a neonatal mouse model of hypoxia-reperfusion injury, AP39 administered intranasally in a liposomal formulation 24 ​h post-injury attenuated neuronal damage and improved histological parameters [137]. A recent study investigating glutamate-induced excitotoxicity following cerebral ischemia in rats (MCAO model) demonstrated that administration of AP39, given 10 ​min after reperfusion, resulted in a dose-dependent reduction of infarct volume and neurological impairment [138]. The study further reported that AP39 modulated key components of glutamate homeostasis by upregulating the expression of the excitatory amino acid transporter GLT-1 and downregulating the vesicular glutamate transporter VGLUT1. These effects contributed to decreased glutamate accumulation in the motor cortex and mitigated excitotoxic neuronal damage during post-ischemic recovery.

AP39 has also been investigated in the context of neurodegenerative diseases, particularly Alzheimer's disease (AD) and Parkinson's disease (PD). In primary neuronal cultures derived from APP/PS1 transgenic mice, low concentrations of AP39 were shown to enhance mitochondrial bioenergetics and improve cell viability [139]. Notably, in the same study, six weeks of AP39 administration led to a significant improvement in spatial memory and a marked reduction in cerebral amyloid-β (Aβ) plaque deposition in APP/PS1 mice. A consistent finding across these studies is that AP39 dampens oxidative stress, suppresses reactive oxygen species (ROS) accumulation, and inhibits caspase-1 and caspase-3 activation, mechanistic hallmarks linked to its observed neuroprotective effects.

A recently developed mitochondria-targeted nanomotor H_2_S donor (PCM), composed of l-cysteine as the H_2_S-releasing moiety, polyethylene glycol (PEG), and a positively charged methacryloyloxyethyl phosphorylcholine component, was reported to achieve efficient blood brain barrier penetration, CBS-dependent H_2_S release, and selective mitochondrial localisation in neuronal cells [140]. In the MPTP-induced mouse model of Parkinson's disease, PCM treatment led to behavioural improvements, supporting its potential as a neuroprotective agent [140].

An alternative approach for mitochondrial H_2_S delivery involves the use of synthetic peptides that direct the attached donor compound specifically to mitochondria [[141], [142], [143]]. A recent study reported the design of a hybrid molecule, JC112, which combines a naturally occurring tripeptide, L-alanyl-L-cystinyl-l-glutamine (ACQ), with a dithiolethione-based H_2_S-releasing moiety ACS48 [144]. In cultured HT22 hippocampal neurons subjected to glutamate-induced excitotoxic stress, JC112 significantly reduced calcium-dependent ROS production, limited the recruitment of apoptosis-inducing factors, and mitigated overall cellular injury, suggesting protective effects in models of neuronal oxidative damage.

Together, these advancements highlight the growing sophistication of mitochondria-targeted H_2_S delivery strategies and their therapeutic relevance across a spectrum of acute and chronic neuropathologies, setting the stage for broader discussions on emerging delivery platforms, systemic modulation, and translational potential in neurodegenerative disease.

### Natural product (Nation donors diallyl disulfide garlic)

**Fig. 5:**
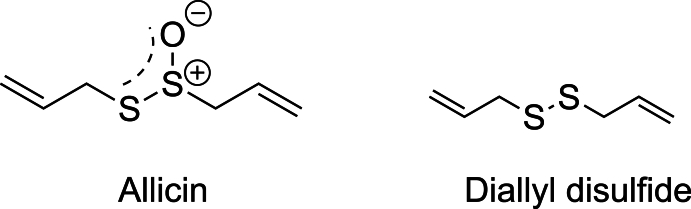
Chemical structures of naturally occurring organosulfur compounds with hydrogen sulfide (H_2_S)-releasing properties. Shown are allicin (S-(prop-2-en-1-yl) prop-2-ene-1-sulfinothioate) and diallyl disulfide (3-[(prop-2-en-1-yl)disulfanyl]prop-1-ene), both derived from Allium species (e.g., garlic) and known to liberate H_2_S through thiol-dependent reactions in biological systems.

**Fig. 6:**
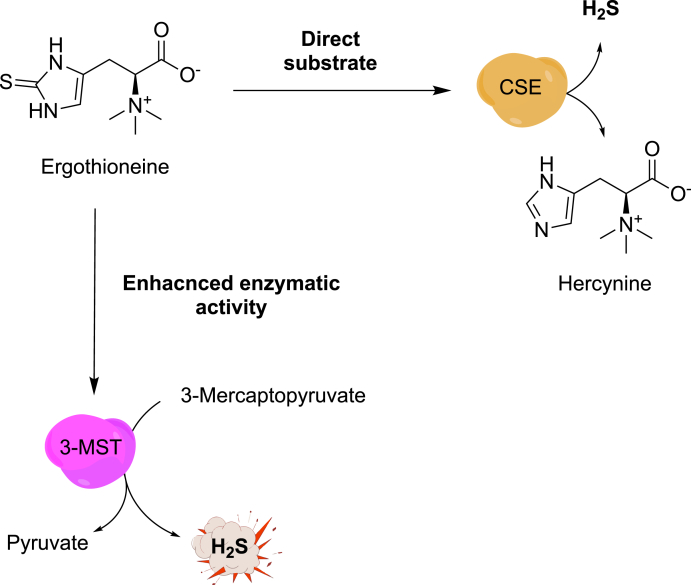
Mechanism of ergothioneine-mediated H_2_S production *via* CSE and 3-MST pathways. Ergothioneine serves as an alternative substrate for cystathionine γ-lyase (CSE), enhancing hydrogen sulfide (H_2_S) generation and promoting widespread protein persulfidation, including activation of cytosolic glycerol-3-phosphate dehydrogenase (cGPDH), which elevates NAD^+^ levels. Additionally, ergothioneine directly activates mitochondrial 3-mercaptopyruvate sulfurtransferase (3-MST), augmenting mitochondrial respiration and improving exercise performance.

Among natural products with emerging neuroprotective potential such as garlic-derived organosulfur compounds diallyl disulfide (DADS) and allicin [145] (Fig. 5), ergothioneine (Fig. 6) has attracted growing attention for its unique antioxidant properties and possible role in mitigating neurodegenerative disease [146]. Ergothioneine ((2S)-3-(2-thioxo-2,3-dihydro-1H-imidazole-4-yl)-2-(trimethylammonio)propanoate; ERG) is a naturally occurring thiol containing amino acid derived from histidine, first isolated in early 19th century from *Claviceps purpurea*, a strain of ergot fungi [147]. Though mammals cannot synthesize it, ergothioneine accumulates in tissues *via* the organic cation transporter OCTN1 highly expressed in animal tissue, suggesting a conserved physiological role. Historically regarded as a dietary antioxidant sourced primarily from mushrooms, fermented beans and red meat, its redox-active properties have attracted growing attention in the context of cellular stress adaptation [148,149]. Recent research has identified ergothioneine as a potent cytoprotective molecule, capable of scavenging reactive oxygen species and mitigating oxidative stress, key processes implicated in neurodegeneration. Its molecular structure containing sulphur atom that exists in the tautomeric equilibrium between thione and thiolate form allows interaction with sulfur-based redox pathways, including potential crosstalk with endogenous hydrogen sulfide (H_2_S) signalling [149]. Two recent parallel studies have provided mechanistic insights demonstrating that H_2_S-synthesizing enzymes (CSE and 3-MST) are key targets mediating the beneficial effects of ergothioneine-derived H_2_S on cellular bioenergetic status *in vivo* [150,151]. Both studies point to ergothioneine acting as a H_2_S-related modulator of mitochondrial enzymes, improving bioenergetic capacity, exercise performance and contributing to lifespan. Emerging evidence suggests that decreased ergothioneine levels correlate with cognitive and functional memory decline and neurodegenerative pathologies such as Alzheimer's and Parkinson's disease [[152], [153], [154]].

A recent investigation demonstrated that ergothioneine (ERG), when administered intranasally, enhanced short-term memory performance in a mouse model of multiple system atrophy (MSA) [155]. In this study, ERG treatment was shown to promote an increase in monomeric α-synuclein expression while concurrently decreasing dimeric α-synuclein levels, ultimately leading to an overall reduction in α-synuclein oligomerisation. These findings indicate that ERG may facilitate the clearance of pathogenic α-synuclein aggregates, supporting its potential as a therapeutic candidate for individuals with MSA. These findings have fuelled interest in its therapeutic potential as a dietary supplement or adjunctive agent aimed at restoring redox balance and protecting mitochondrial function in vulnerable neuronal populations.

Although ergothioneine has been suggested to act, in part, as a sulphur carrier and antioxidant, its pharmacological effects likely involve broader mechanisms. Its clinical translation remains challenging due to its dietary ubiquity and the associated difficulty in controlling background levels in clinical trials, despite its excellent bioavailability *via* the OCTN1 transporter.

### Alternative strategies

In addition to classical H_2_S donors, emerging alternative delivery strategies are being explored to improve the precision, kinetics, and targeting of sulfide-based therapeutics. Among these, metal–organic frameworks (MOFs) have attracted significant attention due to their structural tunability, high loading capacity, and ability to provide controlled, sustained release. MOFs are versatile porous coordination polymers with high surface area, tuneable pore environments, and modular structures, making them promising platforms for gasotransmitter storage and delivery, including H_2_S [156]. Within MOFs, H_2_S coordinates to open-metal sites and is released upon moisture exposure as water molecules displace bound H_2_S, enabling controlled, sustained delivery, particularly suited for transdermal applications [157]. For central nervous system (CNS) targeting, functionalising MOFs with blood–brain barrier (BBB)-penetrating ligands (e.g., transferrin, RVG peptides) or embedding them into liposomal or polymeric hybrid carriers can further enhance delivery efficiency [158,159]. MOF-based H_2_S systems thus offer a rational, tuneable approach for overcoming pharmacokinetic limitations of conventional donors, with potential applications ranging from wound healing to targeted therapies for neurodegenerative diseases.

## Preclinical and clinical evidence in neurological models

As discussed in previous sections, a robust body of animal and cell-based evidence further highlights H_2_S's neuroprotective effects. In Alzheimer's models, chronic administration of NaHS or GYY4137 improved cognition and reduced amyloid pathology. In models of ischemic stroke or traumatic brain injury, H_2_S donors decreased infarct size, edema and neuronal apoptosis. Neuropathic pain models (e.g. chemotherapy-induced pain) have shown amelioration by H_2_S donors. Parkinsonian models (MPTP or 6-OHDA-lesioned rodents) exhibit less dopaminergic degeneration and improved motor function when treated with H_2_S-releasing compounds. Mechanistic studies in these models consistently report reduced oxidative markers, suppressed inflammation (NF-κB, cytokines) and upregulated survival signals (BDNF, PI3K/Akt) under H_2_S treatment. At the cellular level, neurons exposed to oxygen-glucose deprivation or amyloid-β show greater survival with H_2_S supplementation, *via* restored mitochondrial function and antioxidant enzyme activity. Microglial and astrocyte responses are also modulated towards a less inflammatory phenotype by H_2_S.

Clinical observations. Direct clinical data on H_2_S modulation in neurological disease are limited. No H_2_S-specific therapies have yet reached advanced clinical trials for brain disorders. However, associative evidence hints at clinical relevance. For example, Down syndrome patients (trisomy 21) excrete unusually high thiosulfate levels, reflecting elevated CBS activity and H_2_S production. Importantly, many preclinical successes in H_2_S-based neuroprotection have yet to be tested in humans. While earlier H_2_S-releasing NSAIDs such as ATB-346 advanced to clinical trials, subsequent reports of hepatotoxicity have halted further development [160]. Nevertheless, interest in H_2_S-based therapeutics continues, with several companies (e.g., MitoRx Therapeutics, Oxford) exploring novel H_2_S modulators for clinical use. An ongoing study with a strong bioanalytical focus is examining the quantification of H_2_S in human biological samples, investigating whether alterations in circulating H_2_S levels could serve as a biomarker for Alzheimer's disease and age-related cognitive decline (ClinicalTrials.gov ID: NCT05060848). Thus, while translational evidence is still emerging, the preclinical literature strongly supports further clinical exploration of H_2_S and H_2_S-based therapeutics for neurological therapeutics.

## Pharmacokinetics, safety and dosing considerations

The therapeutic application of H_2_S demands careful attention to its unique pharmacological profile. Under physiological conditions, endogenous H_2_S is maintained at very low (nanomolar to low micromolar) concentrations and undergoes rapid metabolism to thiosulfate and sulphate. By contrast, exogenous administration *via* gaseous H_2_S or fast-releasing donor compounds can lead to sharp transient peaks, posing toxicity risks. At high doses, H_2_S can acutely inhibit cytochrome *c* oxidase (Complex IV), an effect that may be condition-dependent and even beneficial, for example, during cardiac ischemia-reperfusion and potentially in stroke, where rapid H_2_S release from MitoPerSulf, a novel mitochondria-targeted donor [161], transiently inhibits Complex IV, which disrupts reverse electron transport (RET)-driven ROS production to mitigate IR-related damage. Conversely, uncontrolled surges of H_2_S can cause respiratory collapse, highlighting the importance of slow-releasing donors for safe *in vivo* application [162]. For instance, it was demonstrated that GYY4137 releases H_2_S in a pH-dependent manner, with peak levels observed in phosphate buffer (pH 7.4) at around 15 ​min, followed by a plateau lasting approximately 75 ​min [162]. However, despite its widespread use in a variety of experimental setups to date, the actual concentration of GYY4137 administered as well as its pharmacokinetic profile, particularly tissue distribution has not been adequately assessed in biological contexts, especially when delivered *via* ad libitum drinking water or intraperitoneal (i.p.) injection. A significant limitation in the field remains the lack of comprehensive studies that addresses drug's absorption, distribution, metabolism, excretion, and pharmacokinetics (ADME/PK studies) for most H_2_S donors. It is unclear whether commonly used compounds such as NaHS or GYY4137 reach pharmacologically relevant concentrations in target tissues such as the brain. The absence of such data complicates the interpretation of therapeutic potential, especially in the context of neurodegeneration where blood–brain barrier permeability is critical. Bioavailability is influenced not only by pharmacokinetics but also by factors such as tissue perfusion and endogenous enzymatic turnover. Interactions with other gasotransmitters further complicate the picture: H_2_S can inhibit phosphodiesterases to elevate cGMP [111] or react with nitric oxide and its precursors or metabolites (e.g., nitrite and *S*-nitrosothiol) to form reactive intermediates such as HSNO and nitroxyl (HNO) [114,163,164]. Future studies must place greater emphasis on rigorous chemical validation, donor specificity, and translational pharmacology to accurately determine the role of H_2_S-based therapeutics.

Safety data in humans remain limited and derive mostly from non-neurological contexts, where low micromolar blood concentrations appear to be well tolerated, though long-term effects remain unclear. Animal studies show wide variation in dosing regimens; for example, mouse models of Alzheimer's disease typically used GYY4137 at 100–200 ​μmol/kg intraperitoneally, yielding cognitive improvements [165,166]. Clinical translation will require optimization of dosing schedules. It is important to acknowledge that many widely used H_2_S donors, including GYY4137 and NaHS, are often applied at supra-physiological concentrations, frequently in the high micromolar to millimolar range raising questions about the translational relevance of observed effects [167,168]. The actual intracellular or tissue concentrations of H_2_S generated under such conditions remain largely undefined due to the lack of rigorous pharmacokinetic (PK) and biodistribution data. As such, interpretations of their bioactivity require careful contextualization, and further studies with precise dosing and quantitative tracking are warranted. In contrast, mitochondria-targeted donors such as AP39 and MitoPerSulf demonstrate biological efficacy at nanomolar concentrations in multiple *in vitro* and *in vivo* models (e.g., worms, fish, mammalian cells), suggesting a more physiologically relevant and potentially translatable approach. These tools exemplify a new generation of H_2_S donors with improved targeting and dosing profiles, aligning better with therapeutic feasibility. Moreover, pharmacological inhibitors of endogenous H_2_S synthesis (e.g., PAG, AOAA) are non-specific and can induce homocysteine accumulation, which is why current therapeutic efforts focus on donor compounds. In summary, although H_2_S diffuses rapidly and has a short biological half-life, advances in donor chemistry and careful dosing strategies offer the potential to achieve neuroprotective concentrations without systemic toxicity.

## Future directions and emerging technologies

Future research is poised to advance H_2_S-based neurotherapeutics on multiple fronts. Chemical innovation will produce donors with tuneable kinetics and targeting: for example, enzyme-triggered donors that release H_2_S only in diseased tissue, or light-activated donors for precise spatiotemporal control. Nanotechnology and biomaterials could enable safe H_2_S delivery to the brain (e.g. H_2_S-loaded nanoparticles or hydrogels that cross the blood–brain barrier). Beyond direct H_2_S storage, metal–organic frameworks (MOFs) can also be designed to encapsulate H_2_S-releasing compounds such as thiol-based donors, dithiolone derivatives, or metal-bound persulfides within their porous architecture, allowing controlled, sustained release profiles responsive to local physiological cues such as acidic pH, elevated glutathione concentrations, or reactive oxygen species. Imaging advances will allow tracking of H_2_S *in vivo*: new fluorescent probes and PET tracers are being developed to measure endogenous and donor-derived sulfide dynamics in real time. On the biological side, genetic tools (CRISPR/Cas9) can create refined models with neuron-specific CBS/CSE knockouts or mutants, clarifying H_2_S's roles. The interplay between microbiome-derived H_2_S and the brain (*via* gut–brain axis) is another intriguing area. Mechanistically, much remains to be learned about H_2_S's epigenetic effects and interaction with other cellular pathways. For instance, how does H_2_S influence histone acetylation or DNA methylation beyond neurons? Can we exploit H_2_S-mediated miRNA circuits (e.g. deliver miR-125b inhibitors) to adjust H_2_S levels? Systems biology and network analyses may elucidate how H_2_S integrates with NO or with recently discovered endogenous pathway responsible for cyanide (CN^−^) production [169,170] in neural signalling. Clinically, early-phase trials might explore repurposing H_2_S-donating drugs for neuroprotection or as adjuvants in stroke/neurodegeneration. In sum, the convergence of novel chemistries, molecular biology and bioengineering will expand our ability to modulate H_2_S safely and effectively in the brain.

## Conclusion

Hydrogen sulfide has emerged from its evolutionary legacy as a primordial gas to a versatile neuromodulator with far-reaching therapeutic potential. As reviewed here, H_2_S participates in the regulation of redox balance, mitochondrial function, synaptic plasticity, and neuroinflammation, all processes central to neurodegenerative disorders and acute neural injuries. Preclinical evidence strongly supports the idea that carefully restoring H_2_S homeostasis can confer neuroprotection, and new delivery technologies, including organelle-targeted donors and natural compounds like ergothioneine, are expanding the therapeutic toolkit. Although clinical translation remains in its infancy, advances in donor chemistry, delivery platforms, and mechanistic understanding suggest that H_2_S-based neurotherapeutics represent a promising frontier for future treatments targeting both chronic neurodegeneration and acute CNS injury.

## Author contributions

J. Lj. M conceived and designed the manuscript and provided intellectual leadership throughout the project. J. L. M and J. J. L drafted the manuscript. J. L. M, J. J. L, R. E. M and J. Lj. M all contributed to manuscript revision, provided critical feedback, and approved the final draft.

## Declaration of generative AI and AI-assisted technologies in the writing process

During the preparation of this work the authors used ChatGPT in order to improve language, correct grammar and readability with caution. After using this tool/service, the authors reviewed and edited the content as needed and takes full responsibility for the content of the publication.

## Declaration of competing interest

The authors declare that they have no known competing financial interests or personal relationships that could have appeared to influence the work reported in this paper.
