# Corrigendum to “Black gas, bright future: H_2_S based therapeutics for neurodegenerative disorders” [Neurotherapeutics Volume 22, (Issue 6) (2025) e00755]

**DOI:** 10.1016/j.neurot.2026.e01052

**Published:** 2026-08-17

**Authors:** Jordan L. Morris, Jordan J. Lee, Russell E. Morris, Jan Lj. Miljkovic

**Affiliations:** a MRC Mitochondrial Biology Unit, Keith Peters Building, Cambridge Biomedical Campus, University of Cambridge, CB2 0XY, UK; b Department of Medicine, University of Cambridge, Addenbrookes Hospital, Hills Road, Cambridge CB2 0QQ, UK; c EaStCHEM School of Chemistry, University of St Andrews, St Andrews, KY16 9ST, UK

The authors regret that, in the published article, Awen Oncology Ltd. was incorrectly listed as Dr Jordan L. Morris's affiliation. The correct affiliation for Dr Jordan L. Morris is: MRC Mitochondrial Biology Unit, Keith Peters Building, Cambridge Biomedical Campus, University of Cambridge, CB2 0XY, UK. This correction concerns only the affiliation information and does not affect the authorship, manuscript content, interpretation, conclusions, or scientific message of the article.

The authors would like to apologise for any inconvenience caused.

